# GPER Function in Breast Cancer: An Overview

**DOI:** 10.3389/fendo.2014.00066

**Published:** 2014-05-06

**Authors:** Rosamaria Lappano, Assunta Pisano, Marcello Maggiolini

**Affiliations:** ^1^Department of Pharmacy, Health and Nutritional Sciences, University of Calabria, Rende, Italy

**Keywords:** GPR30, GPER, estrogen, estrogen receptor, breast cancer

## Abstract

The G-protein-coupled estrogen receptor-1 (GPER, formerly known as GPR30) has attracted increasing interest, considering its ability to mediate estrogenic signaling in different cell types, including the hormone-sensitive tumors like breast cancer. As observed for other GPCR-mediated responses, the activation of the epidermal growth factor receptor is a fundamental integration point in the biological action triggered by GPER. A wide number of natural and synthetic compounds, including estrogens and anti-estrogens, elicit stimulatory effects in breast cancer through GPER up-regulation and activation, suggesting that GPER function is associated with breast tumor progression and tamoxifen resistance. GPER has also been proposed as a candidate biomarker in triple-negative breast cancer, opening a novel scenario for a more comprehensive assessment of breast tumor patients.

## Introduction

Estrogen receptors (ERα and ERβ) belong to the family of ligand-regulated transcription factors that mediate a wide range of hormone-induced physiological responses (1). In addition, estrogen and their cognate receptors are mainly involved in the progression of hormone-sensitive tumors including breast cancer. Consequently, selective estrogen receptor modulators (SERMs) were developed and used for decades in order to suppress estrogen signaling in patients with breast tumor. Tamoxifen, the first SERM approved for the treatment of breast cancer, has evidenced the ability to reduce both breast cancer recurrence and contra-lateral cancer by approximately 40–50% in women with early breast cancer (2). Although tamoxifen has demonstrated the effectiveness in preventing numerous ER-positive breast tumors, no beneficial effects was observed in any of the clinical trials aimed to evaluate the progression of ER-negative tumors (3). As several long-term side-effects were associated with tamoxifen treatment, including an increased risk of endometrial cancer and thromboembolism, additional SERMs like raloxifene were developed in recent years. In this regard, clinical trials have demonstrated that patients receiving raloxifene show a reduced amount of side-effects than tamoxifen-treated women. Nevertheless, raloxifene exhibited a fewer effectiveness than tamoxifen toward the prevention of invasive breast cancer (3). More recently, a third-generation SERM named lasofoxifene has evidenced high efficacy in reducing the incidence of ER-positive breast cancer, along with decreased side-effects compared to tamoxifen or raloxifene treatment (4). However, the use of lasofoxifene has not yet been approved by FDA in the prevention of breast cancer.

To date, an alternative strategy in preventing the progression of ER-dependent breast tumors is represented by drugs that inhibit the aromatase enzyme and interfere with the biosynthesis of estrogens, leading to a drastic reduction of circulating estrogen levels (5). Accordingly, clinical trials have suggested that aromatase inhibitors (AIs) are highly effective in preventing invasive ER-positive breast tumors in high-risk women (6, 7), even though significant side-effects have been observed in follow-up studies (8). The aforementioned observations indicate that SERMs and AIs are not able to prevent the development of ER-negative breast cancer, hence suggesting the need of additional prognostic and predictive factors beyond ERα (9), toward more comprehensive therapeutic strategies particularly in these types of tumors.

17β-Estradiol (E2) triggers rapid effects by activating numerous transduction signaling, including the insulin-like growth factor I receptor (IGF-IR) and members of the epidermal growth factor receptor (EGFR) family (1, 10, 11). Moreover, it has been well recognized the involvement of the G-protein-coupled receptor named GPR30/GPER in rapid responses to both estrogens and anti-estrogens (12). In this regard, it has been suggested that these molecules can induce growth effects through the activation of GPER-mediated signaling in ER-negative breast tumors (13–15). Taking also into account that one in four patients with ER-positive tumors does not respond to anti-estrogens (16), the existence of an alternative estrogen receptor as GPER, may provide the basis for a better understanding of novel mechanisms by which estrogens/anti-estrogens stimulate the proliferation of hormone-sensitive cancer cells, including breast carcinoma. Further supporting these findings, the expression of GPER has been found associated with the development of tamoxifen resistance in breast cancer patients (17–19).

The numerous evidence that underline the complex action exerted by GPER up-regulation and activation in the progression of breast cancer will be summarized here, having also a look at the chance to consider it as a further biological target for innovative therapeutic strategies in breast tumor.

## GPER Signaling in Breast Cancer Cells

The initial studies, which elucidated some of the biological actions exerted by GPER in breast cancer cells, like the activation of rapid intracellular signaling induced by E2 and the ER antagonists tamoxifen and ICI 182, 780 (13, 14, 20), paved the way for a wide number of studies aimed to characterize the molecular mechanisms involved in GPER-mediated biological responses like cancer cell growth, migration, and invasion. For instance, in ER-negative breast cancer cells, the GPER-dependent ERK1/2 activation was shown to be consequent to the Gβγ subunit-dependent transactivation of EGFR, which occurs through the cleavage and the release of heparan-bound EGF (HB-EGF) by metalloproteinases (MMPs) (13). Likewise, the stimulation of adenylyl cyclase and the cAMP-mediated inhibition of the EGFR/ERK pathway was evidenced in rapid responses to estrogenic signals mediated by GPER in ER-negative breast cancer cells (20). Besides, GPER regulates a typical gene signature as also ascertained in a microarray analysis (12, 15). Some of these GPER target genes are involved in the progression of breast malignancies, like *c*-*fos* which is induced by both estrogens and anti-estrogens in ER-negative breast cancer cells through the involvement of the EGFR/MAPK signaling cascade (14, 15, 21, 22). Other genes induced by estrogens and anti-estrogens through the GPER-dependent pathway are the early growth response-1 (*Egr-1*) (15, 23) and the connective tissue growth factor (*CTGF*), which is also up-regulated by insulin-like growth factor I (IGF-I) and hypoxia through GPER (15, 21, 22, 24, 25). Similar results were obtained in cancer-associated fibroblasts (CAFs) derived from breast cancer samples, suggesting that the stimulatory action of GPER may also be elicited through these key players of the tumor microenvironment (15, 26–28). In accordance with these findings, a recent study highlighted the potential of GPER to mediate the production of estradiol in breast CAFs through the activation of the EGFR/ERK signaling (29).

A variety of phyto- and xeno-estrogens are able to stimulate rapid protein kinases activation, cAMP production, and gene transcription in breast cancer cells through GPER, as observed upon estrogen and anti-estrogen exposure (14, 30–35). In this regard, it should be noted that many of the aforementioned compounds exhibit binding properties for both the classical ER and GPER, albeit some ligands exert opposite functions through these receptors. For instance, estriol elicits ERα agonism but GPER antagonism in breast cancer cells (21), conversely OHT acts as ERα antagonist and GPER agonist (12). Therefore, the identification of selective GPER ligands is needed toward a better characterization of the GPER signaling along with the assessment of the specific biological responses mediated by each estrogen receptor subtype. So far, the selective GPER agonists G-1 (36), as well as GPER-L1 and GPER-L2 (37), allowed the evaluation of the potential role played by GPER in breast cancer cells (21, 38, 39). In addition, antagonist ligands of GPER were recently identified, further contributing to a better understanding of GPER action in different cell contexts (19, 22, 40–43). Among these GPER antagonists, the compound named MIBE (22) showed the peculiar property to inhibit both GPER- and ER-mediated signaling. In this regard, it should be highlighted that a complex interplay between GPER and ERα has been involved in gene expression changes toward breast cancer progression (44, 45). Therefore, MIBE could be used as an innovative pharmacological approach in order to target breast carcinomas, which express one or both receptors at the beginning and/or following tumor progression, hence ensuring major therapeutic benefits with respect to the use of ER antagonists.

## GPER-Mediated Biological Functions in Breast Cancer Cells

The ligand activation of GPER signaling along with the up-regulation of certain GPER target genes were involved in the proliferation of breast cancer cells, supporting the opinion that GPER may contribute to breast carcinogenesis (21, 22, 30, 33, 38). Likewise, it has been reported that GPER activation stimulates the migration of breast cancer cells through CTGF (15), cyclin E (43), the notch pathway (46), and the CXC receptor-1 (CXCR1) (47). Furthermore, GPER agonists were shown to promote the invasion of inflammatory breast cancer cells (48) as well as breast cancer cell adhesion through the calcium-dependent cysteine protease (calpain) activation (49), suggesting a potential of GPER to facilitate the progression of metastatic processes. Supporting the potential involvement of GPER in breast cancer progression, its activation lead to certain deformations of breast glandular structure that characterize the malignant transformation of breast tissue (50). Moreover, GPER-dependent proliferation of non-tumorigenic breast epithelial cells was recently assessed, suggesting a role for GPER also in estrogen-induced breast physiology and pathology (51).

The proliferation and migration of breast cancer cells and CAFs were also evidenced upon growth factor- and hypoxia-induced up-regulation of GPER expression (24, 42, 52, 53). Of note, many of these studies have revealed a cross-talk between EGFR and GPER as observed for other GPCRs (25, 45, 52, 54–56). In particular, the ligand activation of the EGFR transduction pathway was shown to trigger GPER expression in both ER-negative and -positive breast cancer cells (45, 54). Collectively, these findings suggested that this mechanism may extend the potential of EGFR to engage estrogenic signals in breast tumor progression. Analogously, EGFR was involved in the up-regulation of GPER expression by hypoxia in breast cancer cells and CAFs, indicating that GPER may also play a role in hypoxia-induced angiogenesis (25, 52). Moreover, the IGF-I was shown to be able to stimulate the expression of GPER through the IGF-IR/PKC/MAPK transduction pathway in breast cancer cells (24). Altogether, the ability of EGFR and IGF-IR ligands as well as hypoxia to regulate GPER expression and function may be included among the molecular mechanisms leading to cell proliferation, migration, tumor angiogenesis that are mainly involved in breast cancer progression.

## GPER in Breast Carcinomas and Its Role in the Tamoxifen Resistance

G-protein-coupled estrogen receptor-1 is widely expressed in breast cancer cell lines and breast primary tumors (13, 15, 54, 57, 58). By using an immunohistochemical-based approach in breast carcinomas, Filardo and coworkers originally demonstrated that the expression of GPER correlates with clinical and pathological biomarkers of poor outcome as HER-2, increased tumor size and metastasis (59). In patients with GPER-positive tumors treated with tamoxifen, GPER expression results increased while the overall survival of patients decreased, contrary to what observed in patients who did not receive tamoxifen (18). Likewise, it has been recently reported that GPER overexpression and plasma membrane (PM) localization represent crucial events in breast cancer progression as well as the absence of PM GPER was found associated with long-term prognosis in tamoxifen-treated primary breast cancer (60). On the basis of these data, it could be argued that the treatment with tamoxifen in breast cancer patients expressing high levels of GPER should be carefully evaluated.

A number of *in vitro* studies suggested that targeting GPER signaling as well as interfering with the up-regulation of GPER may be a potential strategy to hamper the resistance to tamoxifen-based endocrine therapy in breast tumors. The initial evidence on the ability of OHT to exert agonistic activity toward GPER in various cancer cell lines, including breast carcinoma (14, 34, 61, 62) suggested that classical anti-estrogenic agents may stimulate rather than inhibit a subset of tamoxifen-resistant tumors. In this regard, it should be pointed out that in endometrial cancer cells, GPER mediates biological responses not only to tamoxifen but also other SERMs like raloxifene and the ER antagonist ICI 182,780 (63), hence revealing an additional mechanism which may be involved in the increased risk of endometrial cancer in patients treated with these compounds. Moreover, EGFR ligands were shown to up-regulate GPER expression by activating the EGFR/ERK transduction pathway in ER-positive tamoxifen-resistant breast cancer cells, indicating that the activation of EGFR signaling may contribute to tamoxifen resistance at least in part by up-regulating GPER expression (45). In accordance with these findings, estrogen stimulation of tamoxifen-resistant breast cancer cells led to the up-regulation of GPER, which in turn increased the cell sensitivity and responses to GPER agonists (17). Further corroborating these data, the GPER antagonist G-15 was recently shown to improve the response to endocrine treatment in tamoxifen-resistant xenografts (19).

## GPER in Triple-Negative Breast Cancer Cells

Approximately, 15–20% of all breast carcinomas are included in the subgroup of triple-negative breast cancer (TNBC) that are characterized by the lack of ERα, progesterone receptor (PR), and EGFR 2 (Her-2) (64). TNBCs, which include diverse subtypes with high levels of molecular heterogeneity, affect younger women and display aggressive biological features, a higher rate of recurrence, and a worse clinical outcome with respect to other breast cancer types (8, 65, 66). As well-defined clinical targets are still lacking, the standard chemotherapy remains the treatment option for women with TNBC, even though promising agents are currently under evaluation in prevention trials (8).

Recently, GPER has been evaluated as a candidate biomarker and putative mechanism for growth regulation of TNBCs. In particular, the knockdown of GPER expression was shown to prevent the proliferation of TNBC cells as well as the EGFR activation and *c*-*fos* expression induced by E2 and OHT (67). Next, a potential role elicited by GPER in TNBCs was suggested by a retrospective analysis demonstrating that GPER is prevalent in TNBCs, associated with young age and possible malignant recurrence (68). Taken together, these data suggest that the inhibition of GPER might be an appropriate targeted therapy in TNBC; however, future studies are needed to further corroborate the above-mentioned findings.

## Controversies and Concluding Remarks

Controversies still exist on the localization of GPER and its function, in particular the potential action as pro-apoptotic mediator. Although GPER belongs to a cell surface receptor family, which conventionally mediates transmembrane signaling of membrane-impermeable ligands, numerous studies demonstrated that GPER is detectable at the PM or intracellular levels in breast cancer cells (54, 58, 63, 69, 70). Interestingly, it was demonstrated that GPER localizes within the nucleus of breast CAFs through a translocation mechanism that is regulated in an importin-dependent manner (26, 27). These findings are in line with other observations regarding many GPCRs, which were detected into diverse cellular compartments (71–73). Although further studies are required to better understand the role played by GPCRs in relation to their localization, the subcellular detection of GPER may be involved in its downstream signaling activity. For instance, it has been recently demonstrated that the different localization of GPER could reflect distinct biological features of breast tumors (74). In particular, cytoplasmic GPER was associated with non-ductal histologic subtypes of breast cancer, low tumor stage, and better histologic differentiation, whereas nuclear GPER was associated with poorly differentiated carcinomas and triple-negative subtypes (74).

A further debate on GPER regards its potential action to interfere with the progression of breast cancer. For instance, the phenolic compounds contained in olive oil named oleuropein and hydroxytyrosol, were recently identified as GPER inverse agonists displaying the ability to inhibit the G-1-induced proliferation of ER-negative breast cancer cells (75). A long-term ERK1/2 activation was proposed to explain at least in part the apoptotic effects induced by oleuropein and hydroxytyrosol, although further mechanisms should be taken into account as the GPER silencing did not completely abrogate the action of oleuropein on cell viability (75). Likewise, previous studies evidenced the capability of G-1 to abolish the growth of breast cancer cells activating pro-apoptotic signals (39, 76–78). In this context, it should be mentioned that in MDA-MB 231 breast cancer cells lacking or minimally expressing GPER (13, 14, 57), the treatment with 2 μM G-1 led to a suppression of cell growth, which persisted also using the GPER antagonist G-15 (79). In accordance with these findings, G-1 suppressed the proliferation of ovarian cancer cells without the involvement of GPER, thus evidencing pro-apoptotic properties that could be elicited in a GPER-independent manner (80).

Further controversial observations have been reported on the functional role exerted by GPER in the reproductive system, particularly in mouse models. For instance, GPER has been involved in stimulating uterine epithelial proliferation in mice (40). However, GPER knockout mice did not displayed developmental or functional defects in the reproductive organs (81, 82). Moreover, G-1 treatment did not trigger proliferative responses in mammary gland and endometrium in wild-type mice (82). Conversely, GPER has been shown to regulate meiotic arrest in oocytes of the Atlantic croaker and zebra fish (83, 84), the proliferative and apoptotic pathways involved in spermatogenesis (44, 85, 86) during male reproductive development, the estrogen-induced stimulation of primordial follicle formation in the hamster ovary (87).

Despite the controversies regarding the role of GPER in breast cancer, the *in vitro* and *in vivo* data available as well as the epidemiological studies have ascertained that GPER may act as an estrogen receptor in different pathophysiological responses, including breast cancer. GPER regulation and functions have also been involved in the resistance to tamoxifen in breast tumor patients, thus therapeutic approaches targeting the GPER-mediated signaling may be taken into account in setting innovative pharmacological strategies, in particular to hamper the failure of classical anti-estrogens in breast malignancy. The identification of further molecules targeting both estrogen receptor types is strongly required to effectively reduce breast cancer incidence and recurrence.

## Conflict of Interest Statement

The authors declare that the research was conducted in the absence of any commercial or financial relationships that could be construed as a potential conflict of interest.
